# Characterization of copy number alterations in a mouse model of fibrosis‐associated hepatocellular carcinoma reveals concordance with human disease

**DOI:** 10.1002/cam4.606

**Published:** 2016-01-18

**Authors:** Grace Chappell, Grace O. Silva, Takeki Uehara, Igor P. Pogribny, Ivan Rusyn

**Affiliations:** ^1^ Department of Veterinary Integrative BiosciencesTexas A&M UniversityCollege StationTexas77843; ^2^ Department of Environmental Sciences and EngineeringUniversity of North CarolinaChapel HillNorth Carolina27599; ^3^ Department of GeneticsUniversity of North CarolinaChapel HillNorth Carolina27599; ^4^ Curriculum in Bioinformatics and Computational BiologyUniversity of North CarolinaChapel HillNorth Carolina27599; ^5^ Lineberger Comprehensive Cancer CenterUniversity of North CarolinaChapel HillNorth Carolina27599; ^6^ Division of Biochemical ToxicologyNational Center for Toxicological ResearchUnited States Food and Drug AdministrationJeffersonArkansas72079

**Keywords:** Chromosomal instability, cirrhosis, copy number alterations, fibrosis, hepatocellular carcinoma

## Abstract

Hepatocellular carcinoma (HCC) is a prevalent human cancer with rising incidence worldwide. Human HCC is frequently associated with chronic liver inflammation and cirrhosis, pathophysiological processes that are a consequence of chronic viral infection, disturbances in metabolism, or exposure to chemical toxicants. To better characterize the pathogenesis of HCC, we used a human disease‐relevant mouse model of fibrosis‐associated hepatocarcinogenesis. In this model, marked liver tumor response caused by the promutagenic chemical *N*‐nitrosodiethylamine in the presence of liver fibrosis was associated with epigenetic events indicative of genomic instability. Therefore, we hypothesized that DNA copy number alterations (CNAs), a feature of genomic instability and a common characteristic of cancer, are concordant between human HCC and mouse models of fibrosis‐associated hepatocarcinogenesis. We evaluated DNA CNAs and changes in gene expression in the mouse liver (normal, tumor, and nontumor fibrotic tissues). Additionally, we compared our findings to DNA CNAs in human HCC cases (tumor and nontumor cirrhotic/fibrotic tissues) using publicly available data from The Cancer Genome Atlas (TCGA). We observed that while fibrotic liver tissue is largely devoid of DNA CNAs, highly frequently occurring DNA CNAs are found in mouse tumors, which is indicative of a profound increase in chromosomal instability in HCC. The cross‐species gene‐level comparison of CNAs identified shared regions of CNAs between human fibrosis‐ and cirrhosis‐associated liver tumors and mouse fibrosis‐associated HCC. Our results suggest that CNAs most commonly arise in neoplastic tissue rather than in fibrotic or cirrhotic liver, and demonstrate the utility of this mouse model in replicating the molecular features of human HCC.

## Introduction

Hepatocellular carcinoma (HCC) is a prevalent and life‐threatening human cancer 1. Importantly, the incidence of HCC continues to increase, while the incidence and death rates of many other cancers are steadily declining 1, 2. In humans, the majority (70–90%) of HCC cases are concomitant with advanced liver fibrosis or cirrhosis 3. The incidence of HCC in noncirrhotic liver is estimated to be approximately 15–20% of all HCC cases, and only 10–12% in healthy livers. Liver fibrosis and cirrhosis associated with HCC are typically the consequence of toxic insults, disturbances in metabolism, and/or viral infection 4.

HCC is a complex and heterogeneous disease, and while the histopathological features of HCC are well established, the molecular mechanisms that drive the main etiological factors, including cirrhosis‐causing factors, are not well characterized 5, 6. Most human cases of HCC develop in a background of fibrosis or cirrhosis; therefore, characterizing the cellular and tissue features in which tumors develop is as important as identifying the specific genes and pathways that are involved in tumorigenesis. Thus, mouse models that emulate human liver diseases that lead to HCC offer great value in the study of hepatocellular carcinogenesis 7. In addition, understanding the molecular mechanisms that promote pathogenesis and progression of HCC will contribute to effective prevention, as well as the development of new therapies 8.

Many mouse models of HCC have been previously developed and demonstrate varying levels of similarity to human disease 7. A commonly used mouse liver cancer model is a single low‐dose injection of the genotoxic carcinogen *N*‐nitrosodiethylamine (DEN) into 14‐day‐old male B6C3F1 mice 9. Additionally, repeat dosing with the profibrogenic agent carbon tetrachloride (CCl_4_) results in the development of HCC 10. The combination of genotoxic, for example, DEN, and nongenotoxic, for example, CCl_4_, insults has been used to emulate the comorbidity features often observed in human HCC patients, and offers a relevant model to study the mechanisms involved in hepatocellular carcinogenesis 11. Using this mouse model, we found that epigenetic alterations indicative of genomic instability were prevalent in both tumors and nontumor fibrotic tissues of mice treated with DEN+CCl_4_ compared with liver tissue of vehicle‐treated control animals 12. Chromosomal instability, a condition that results in DNA copy number alterations (CNAs), can occur simultaneously with and promote genomic instability 13, one of the “hallmarks of cancer” 14. Therefore, we hypothesized that CNAs are frequent in mouse liver tumors that developed in fibrotic tissue. Furthermore, due to numerous pathological features shared between human HCC and tumors arising in this mouse model, we posit that many CNAs are shared (i.e., conserved) between both species.

An association between changes in DNA copy number values and the development and progression of cancer has been observed in various types of cancer, in mouse models, and in human clinical samples 6, 15, 16, 17, 18. These CNAs are thought to represent a type of oncogenic driver in the progression of cancer 19; for example, sites of DNA copy number gains are known to harbor oncogenes, while sites of DNA copy number losses include tumor suppressor genes 15, 16. Somatic DNA alterations are frequent in cancer, and recurrent CNAs represent one type of genetic aberration commonly involved in many types of cancer 20. CNAs are associated with complex diseases by various molecular mechanisms, including gene dosage, gene disruption, and gene fusion 21, 22. CNAs are frequently observed in clinical cases of HCC 6, 16, 23, and studies have shown similarities in the frequency and recurrence of CNAs in mouse and human HCC 17. However, the exact mechanistic role of DNA CNAs in carcinogenesis remains unclear.

In this study, publicly available data from human HCC patients and data collected from a controlled in vivo mouse experiment were utilized in the analysis of CNAs. To this end, the translational approach enabled (1) the characterization of chromosomal instability in HCC in a background of liver fibrosis, (2) the identification of genes within copy number‐altered segments that overlap between clinical and experimental conditions, and (3) the association of transcriptional response with DNA CNAs.

## Materials and Methods

### Animals

The in‐life portion of this study, mouse treatments, tissue collection protocols, and incidence of neoplastic liver lesions are detailed in Uehara et al. 11. The exact protocol for the mouse model of fibrosis‐associated hepatocarcinogenesis is detailed in Uehara et al. 24. Those studies were conducted with approval from the Institutional Care and Use Committee at the University of North Carolina at Chapel Hill. In the present work, analyses were conducted using frozen liver (normal, tumor and nontumor fibrotic tissues) samples collected from male B6C3F1/J mice injected *i.p*. with DEN (1 mg/kg) in sterile phosphate buffered saline (PBS; 15 mL/kg) at 2 weeks of age, followed by *i.p*. injections with CCl_4_ (0.2 mL/kg) diluted in sterile olive oil two times per week for an additional 14 weeks beginning at 8 weeks of age. Control mice were injected with sterile PBS and sterile olive oil only. All mice were killed at 22 weeks of age.

### DNA extraction and array comparative genomic hybridization

Genomic DNA was extracted from frozen liver samples from vehicle control and DEN+CCl_4_‐treated mice using a DNEasy kit (Qiagen, Valencia, CA). Eighteen mouse tumor samples were included in the study, as well as 18 matched nontumor samples taken from fibrotic, nontumorous, surrounding liver tissue. Liver DNA from 6 vehicle control mice was pooled and used as the reference genome in the aCGH experiments. Genomic DNA was prepared and hybridized to a SurePrint G3 4 × 180K Mouse Genome CGH Microarray (Agilent Technologies, Santa Clara, CA), which included content sourced from the UCSC mm9 (NCBI Build 37), according to the manufacturer's instructions and a 2‐color/sample strategy.

### Normalization and segmentation of array data

For the mouse copy number data, all mouse tumor samples were assayed versus the pooled liver DNA from vehicle control mice and the R/G ratio was obtained, which is the relative measure of DNA copy number. The R/G ratios were lowess (locally weighted scatterplot smoothing) normalized, followed by segmentation using SWITCHdna 15.

For the human copy number data, publically available circular binary segmented (CBS) 25 copy number data obtained from Affymetrix 6.0 SNP arrays were downloaded through The Cancer Genome Atlas (TCGA) data portal (https://tcga-data.nci.nih.gov/tcga/). The following inclusion criteria were applied to select the samples for the analysis: (1) a diagnosis of either cirrhosis or fibrosis as detailed in the pathology report of the patient and (2) available circular binary segmented copy number data for both a tumor and a nontumor liver specimen.

### Identification of group‐specific CNAs and species‐conserved CNAs

To identify and highlight frequently occurring CNAs within an assigned group, SWITCHdna 15 implements a postsegmentation plotting function. Specifically, segmented data from samples within an assigned class (mouse tumor, mouse matched nontumor, human fibrosis‐associated tumor, human cirrhosis‐associated tumor, and human matched nontumor) were titled to highlight the overlapping gain and loss segments, and the frequency of overlapping altered genomic regions was calculated relative to all samples within the group. Group‐specific and conserved CNAs were identified using the add‐on script to SWITCHdna, called SWITCHplus (https://genome.unc.edu/SWITCHplus/) 18. SWITCHplus uses the identified copy number–altered segments from SWITCHdna and/or any other copy number change point detection tools, species‐specific genomic annotations, and categorical information (e.g., tissue type or disease state) to identify frequent CNAs that are significantly associated with a group (e.g., tumors) compared against all other samples (e.g., nontumor liver). Conserved CNAs are identified by SWITCHplus using gene‐level copy number data and remapping of orthologous genes (i.e., mouse genes remapped in human genomic order) to highlight overlapping features (i.e., CNAs that occur at a high frequency and are altered in the same direction of copy number gain or loss). This analysis was conducted in tumor and matched nontumor samples in both humans and mice, as well as between tumors from patients with HCC and cirrhosis versus tumors from patients with HCC and fibrosis.

### Gene expression

Total RNA was extracted from frozen tumor and nontumor fibrotic liver from mice treated with DEN+CCl_4_, as well as normal liver of vehicle control mice using a Qiagen RNeasy kit according to the manufacturer's instructions (Qiagen). RNA was reverse‐transcribed (Applied Biosystems, Carlsbad, CA) and gene expression was determined by quantitative reverse‐transcription PCR (qRTPCR) using gene expression assays (Applied Biosystems). We selected a representative set of genes that were present in copy number–altered segments in tumors from DEN+CCl_4_‐treated mice and in CNA segments in tumors from human cirrhosis patients, as identified by SWITCHplus. From this list of common CNA‐associated genes, a subset of genes known to be associated with hepatocellular carcinogenesis (*Akt1*,* Map3k6*,* Rara*,* Tnf*,* Vegfa*,* Wnt1*,* Wnt10b*,* Tgfb1*, and *Tgfbr2*) or playing a key role in pathways associated with HCC (*Erbb2*,* Jrk*, and *Tgfbr1*) or DNA damage and repair response (*Gadd45b* and *Xrcc1*) were selected for expression analysis. All genes and primers are listed in Table S1. Reactions were performed in a 96‐well assay format using a 7900HT Fast Real‐Time PCR System (Applied Biosystems), and each sample was run in duplicate. The mRNA level of the housekeeping gene *Gusb1* was evaluated in tandem with the experimental runs, and the relative amount of each mRNA transcript was determined using the 2^−ΔΔCt^ method 26. Differences in expression between liver tissue of control mice and tumors from DEN+CCl_4_‐treated mice were evaluated by an unpaired Student's *t*‐test using GraphPad Prism 5 software (San Diego, CA), followed by false discovery rate (FDR) correction. Results are presented as mean ± SD. FDR‐corrected *P* < 0.1 was considered significant.

Human RNA‐seq data were downloaded from TCGA (http://cancergenome.nih.gov/). Genes that were differentially expressed in the tumors relative to matched nontumor samples from cirrhosis patients were identified using the R package DESeq2 27, with Benjamini–Hochberg‐corrected *P* < 0.1 considered significant. Gene Set Association Analysis for RNA‐Seq (GSAASeqSP) 28 was used to identify enriched genesets among the differentially expressed genes in human HCC.

## Results

### Identification of genomic segments with CNAs in mouse fibrosis‐associated tumors

**Figure 1:**
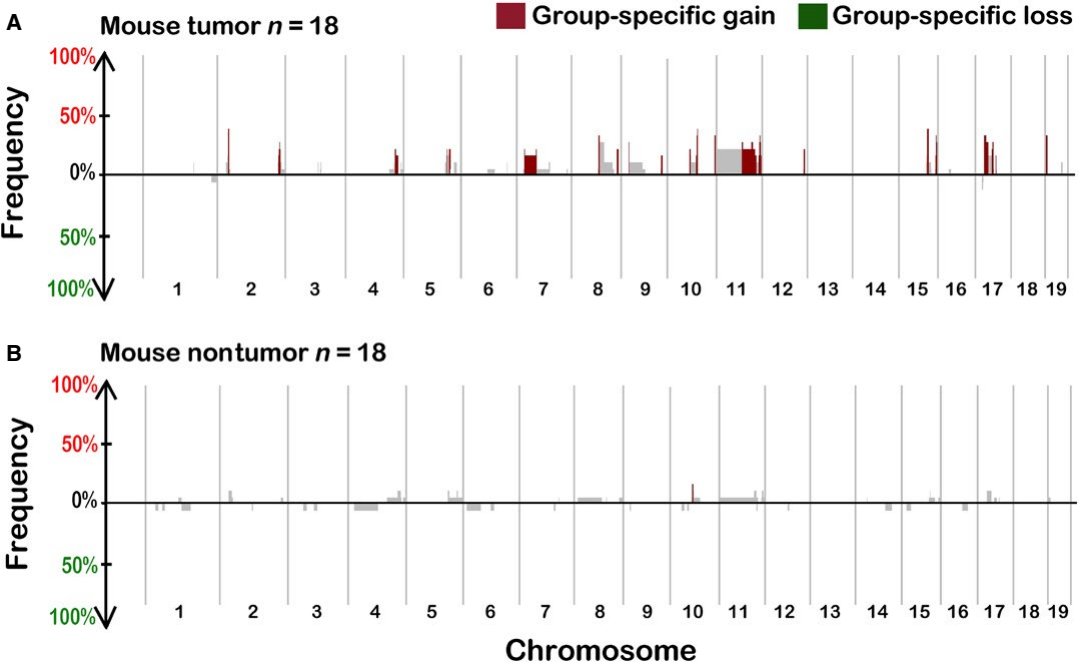
SWITCHplus plots of mouse tumor (A) and matched nontumor fibrotic liver samples (B). The above plots show the frequency of regions with copy number alterations (CNAs) among the sample set of 18 mice, plotted in genomic order. CNAs were more frequent in tumors (top panel) than in samples from matched fibrotic liver tissue. Regions that are significantly different between the two types of tissue and also appear at a frequency of at least 15% are highlighted.

The first objective of this study was to characterize CNAs in both liver tumors and nontumor fibrotic liver tissue in a mouse model of fibrosis‐associated HCC 20. We found that CNAs were much more frequent in tumors (Fig. 1A) than in surrounding fibrotic liver tissue (Fig. 1B). Copy number gains were predominant in the tumors, a pattern that is common in other tumor types 29, while both gains and losses were observed (albeit it at very low frequencies) in the nontumor tissues. Several segments of copy number gain were identified as specific to the tumor samples (segment copy number altered at a frequency of ≥15% and contain at least one gene with a *P*‐value < 0.05, highlighted as “group‐specific” segments in Fig. 1A). In contrast, only one such segment was found in the nontumor tissue on Chr 10 (Fig. 1B).

### CNAs in human HCC associated with cirrhosis or fibrosis

**Figure 2:**
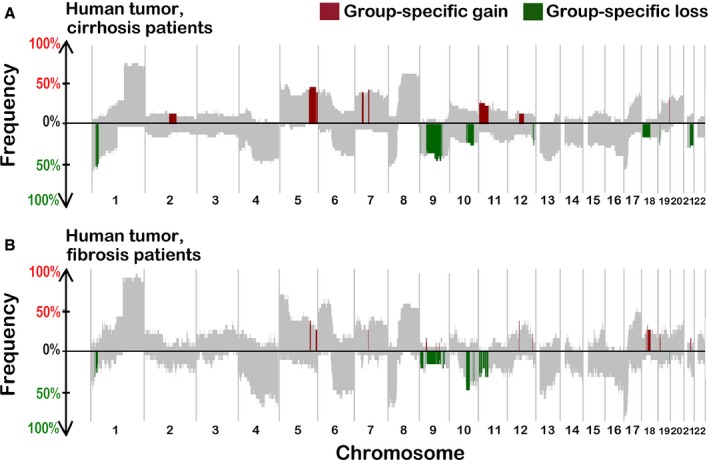
SWITCHplus plots of liver tumors from HCC patients with cirrhosis (A) or fibrosis (B). Regions that are significantly different between the two groups and also appear at a frequency of at least 15% are highlighted.

Another objective of our study was to compare CNAs in the mouse fibrosis‐associated tumors with those observed in human cirrhosis‐ or fibrosis‐associated HCC. Copy number data from 49 human HCC samples (30 patients with cirrhosis and 19 patients with fibrosis) were analyzed. The overall CNA profile of the tumors that arose in either cirrhotic (Fig. 2A) or fibrotic (Fig. 2B) livers was similar, but not identical. Of the chromosomal gains and losses found in our analysis, many are known to be frequent in HCC, such as loss of 1p, 8p, and 17p and gain of 1q, 6p, 8q, and 20q 30; however, we show that these effects are independent of whether HCC arises in the background of fibrosis or cirrhosis. Matched nontumor (cirrhotic or fibrotic) liver samples were available for a subset (*n* = 16) of these 49 patients. In these nontumor tissues, no frequent (present in at least 15% of the samples) copy number‐altered segments were identified (data not shown), similar to our observation in the mouse model (Fig. 1B).

To identify segments with CNAs that were specific either to fibrosis‐ or cirrhosis‐associated HCC in human patients, copy number data were analyzed in the same manner as in the mouse comparative analysis between tumor and nontumor DNA. Several segments were significantly different between these groups (highlighted as “group‐specific” segments in Fig. 2). The segments that were identified as specific to fibrosis‐ or cirrhosis‐associated HCC varied in size and position and, thus, potentially affected genes that were colocated in neighboring genomic regions, which may be particularly prone to chromosomal instability.

### Cross‐species comparative analysis

**Figure 3:**
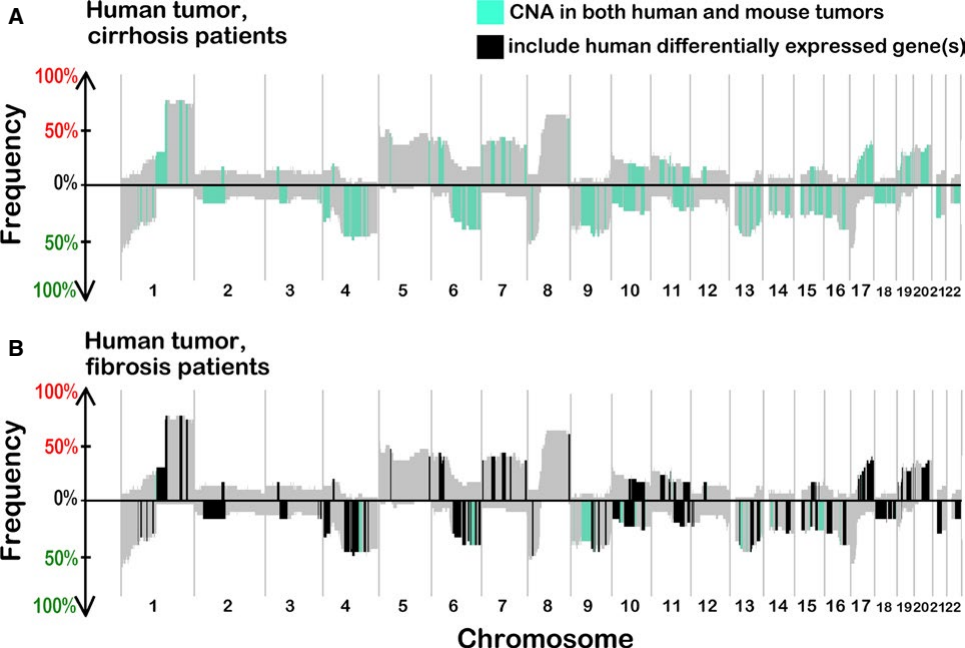
(A) Comparative analysis of human and mouse copy number alterations (CNAs) with homologous genes: mouse segments remapped in human genomic order and compared to all gains/losses in tumors from human cirrhosis patients. Segments with CNAs that include homologous genes between groups (human cirrhosis and mouse tumor) are highlighted. (B) Segments containing genes present in a segment in at least 15% of the tumor samples from human patients with cirrhosis and also present in a mouse tumor segment and that had significantly different mRNA levels (*q* < 0.10) are highlighted. The background SWITCHplot is that of human cirrhosis‐associated tumor data.

Next, the mouse and human CNAs were compared to identify conserved segments between the mouse and human HCC (Fig. 3A). For this analysis, we focused on human cirrhosis‐associated HCC because the CNAs profile was largely similar between fibrosis‐ and cirrhosis‐associated tumors. Fifty‐one percent of copy number‐altered segments observed in the mouse tumor DNA contained at least one gene that was also present in a segment observed at a frequency of 15% or greater in the human tumor samples from cirrhosis patients with HCC (green segments in Fig. 3A). Of all the genes in these segments, one third (33%) was common between mouse and human HCC.

### Gene expression analysis

**Figure 4:**
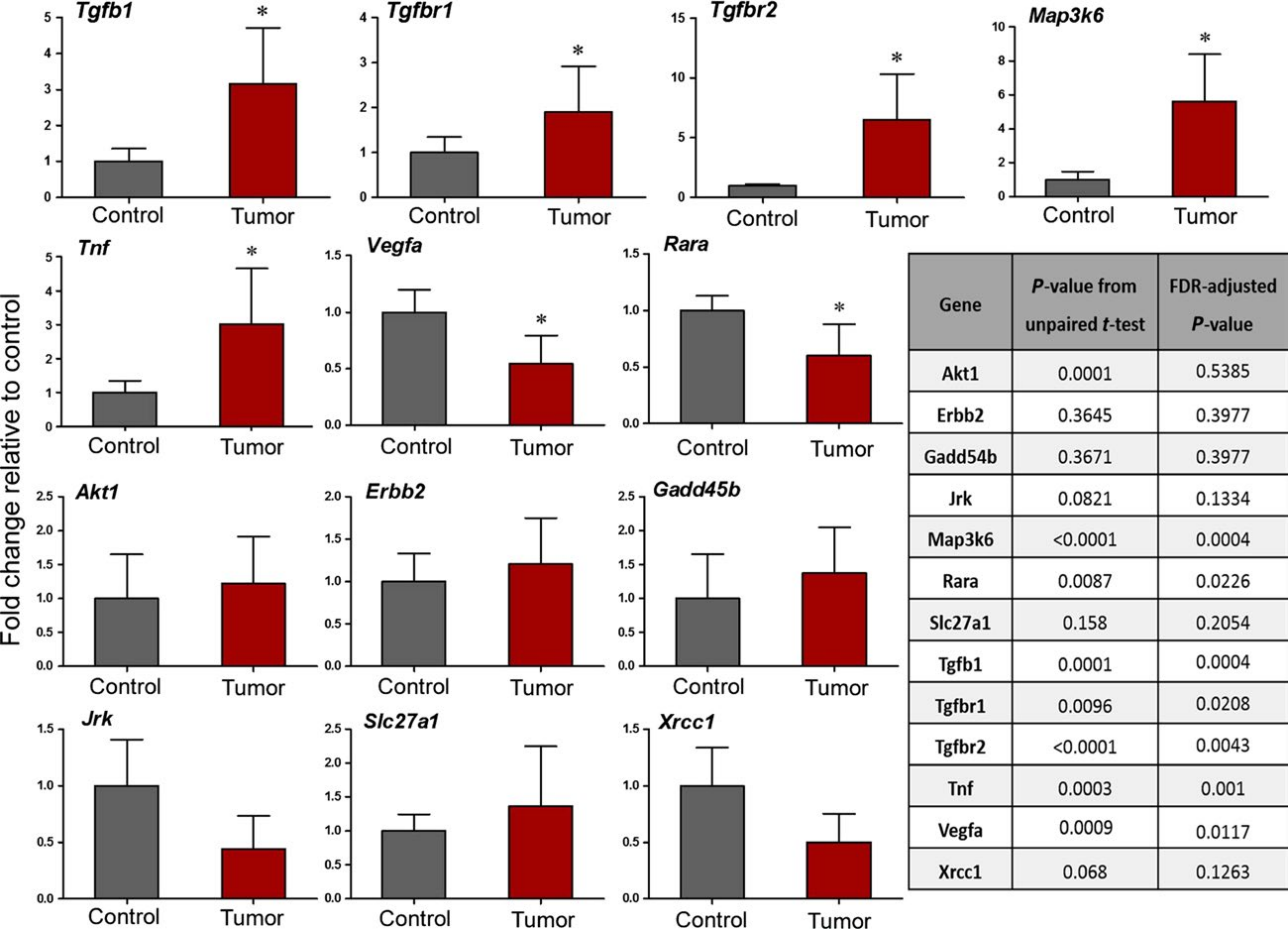
Expression of a subset of genes located in segments of copy number gain in fibrosis‐associated HCC in mice. mRNA level is shown as fold change relative to vehicle control mice, as evaluated by qRTPCR. Results are presented as mean ± SD,* n* = 5 for control group, *n* = 18 for tumor samples. *FDR‐corrected *P*‐value < 0.10.

To evaluate the relationship between CNAs and gene expression, transcript abundance was compared for a set of genes that were found to be gained in copy in both human and mouse liver tumors. For the mouse study, we selected 13 genes to test, based on their location in segments of copy number gain in mouse and human in our study, as well as their known association with carcinogenesis 31. Of these genes, 5 had an increased level of mRNA compared to that from control mice, 2 were down‐regulated, and 6 were not affected (Fig. 4). It is worth noting that the expression of two additional genes tested but not shown, *Wnt1* and *Wnt10b*, was detectable in the mouse tumor tissues, but was below the limit of detection in the controls; thus, while the relative analysis was not possible, these genes were also up‐regulated. In contrast, when expression of the affected genes was tested in nontumor fibrotic mouse liver tissue, no change in expression relative to the controls was observed (data not shown).

**Figure 5:**
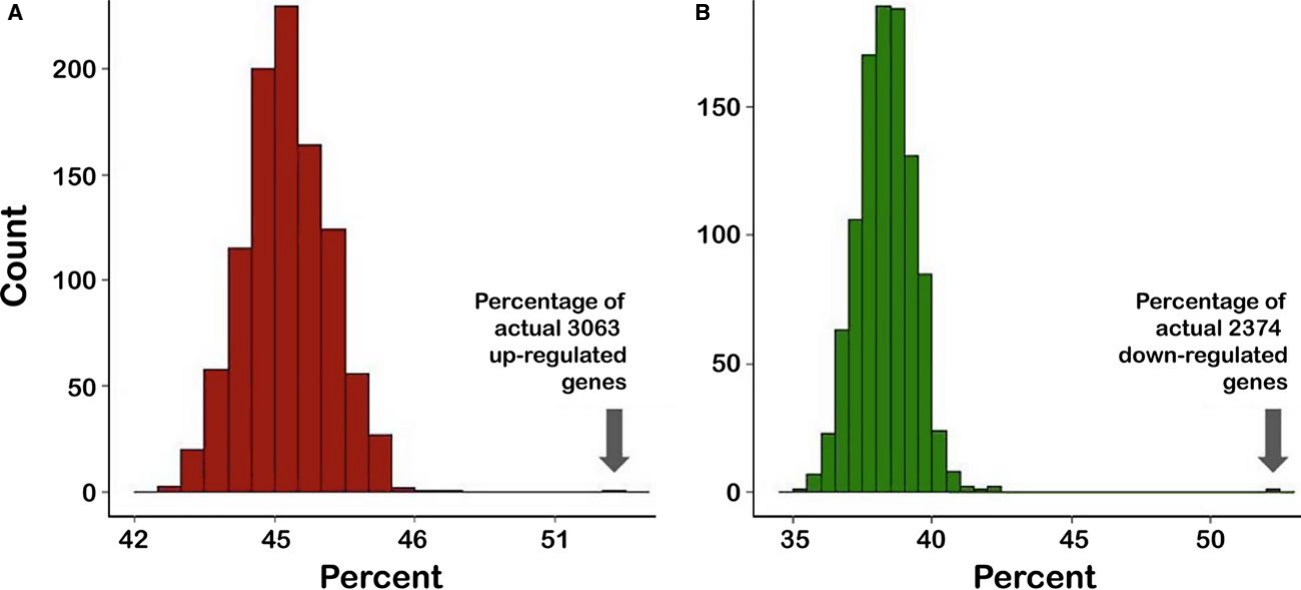
Histograms of the percentage of genes that are present in segments of copy number gain (A) or loss (B) over 1000 permutations of all of the genes expressed in the human HCC patients. The randomly permuted gene set sizes were 3063 and 2374, which are the true numbers of significantly up‐ and down‐regulated genes, respectively. Arrows highlight the percentage of differentially expressed (FDR‐corrected *P*‐value < 0.10.) genes that are also within copy number alterations segments in the concordant direction.

In human cirrhosis‐associated tumor samples, 52% of the significantly up‐regulated (FDR‐corrected *P* < 0.1) genes were also present in segments of gain, and 52% of the significantly down‐regulated genes (FDR‐corrected *P* < 0.1) were located in segments of copy number loss (Fig. 3B). To evaluate the significance of these findings relative to the expected outcome of a randomly chosen set of genes among all expressed transcripts (17,021), we conducted a permutation‐based analysis with the gene set sizes corresponding to the number of significantly down‐ and up‐regulated genes (2374 and 3063, respectively). The distributions of the percentage of genes that fall into a segment of copy number gain or loss over 1000 permutations are shown in Figure 5. Based on a z‐score derived from the distribution of the permutated percentages, we found that there is zero likelihood that the observed concordance between CNAs and the corresponding gene expression changes in human HCC was due to chance (Fig 5).

In addition, we identified enriched genesets among all of the differentially expressed genes in human tumors that were also present in CNA segments in the associated direction (up‐regulated genes in gained segments and down‐regulated genes in loss segments), as well as among all differentially expressed genes (regardless of association with a CNA segment). We found very similar enriched genesets among all genes, compared with enriched genesets among the subset of genes that were found within a copy number altered segment. For up‐regulated genes, the most significant enriched genesets were associated with cell cycle processes and mitosis. For down‐regulated genes, the most significant enriched genesets included immune and defense responses, as well as inflammatory response and receptor activity (Table S2).

To determine the degree of conservation in affected segments between species, we examined whether differentially expressed genes in human HCC have homologs in the genomic regions of gain or loss in mouse HCC. We found that among the genes significantly (FDR‐corrected *P* < 0.1) up‐ or down‐regulated in human cirrhosis‐associated tumors, 30.4% and 22.0% were conserved in the segments that were gained or lost, respectively, in a concordant manner in both mouse and human tumors. The shared percentages of each of the above‐described comparisons are detailed in Table 1, as well as the percentage of genes that were differentially expressed in the “opposite” direction of the associated copy number altered segment.

**Table 1:** Percent of differentially expressed genes (FDR‐corrected *P* < 0.1) that are located in copy number altered segments, respective of direction of gene expression and DNA copy number

CNA type	Gene up‐regulation (%)	Gene down‐regulation (%)
Human copy number gain	52.0	18.2
Human copy number loss	30.1	52.0
Species‐conserved copy number gain	30.4	0.8
Species‐conserved copy number loss	12.8	22.0

## Discussion

Hepatocellular carcinogenesis is a complex process that is the consequence of multiple molecular events that lead to initiation, promotion, and progression 5, 6, 8. The importance of a distinct set of events that are required for carcinogenesis has been emphasized 14, 32, and distinct cellular capabilities that enable tumorigenesis, or “hallmarks of cancer,” are increasingly recognized as essential processes in carcinogenesis 33. To gain a comprehensive perspective of the mechanisms involved in specific cancer types, as well as various etiologies within a type of cancer, investigation of underlying molecular factors of cancer should consider genetic aberrations, epigenetic alterations, changes in gene expression, and CNAs. While many CNAs have no apparent influence on phenotype, others have been definitively linked with disease, including cancer 34. Furthermore, structural and numerical chromosomal changes occur in the majority of cancer cells 35.

An important consideration of the multistep process of carcinogenesis is that the standard mutation rate cannot explain the extensive number of mutations present in many types of cancer cells 36, 37, 38. The acquisition of some form of chromosomal instability is likely a necessary event in tumor development that occurs relatively early and presents an explanation of the numerous karyotypic aberrations that are often observed in malignant tumors 17. The subsequent process of clonal expansion by a stepwise accumulation of numerical chromosome changes would, at least in some cases, be expected to give rise to subpopulations of neoplastic cells related by the same karyotypic abnormalities 39.

Our study provides additional mechanistic information on the role of CNAs in HCC, both in human and mouse disease associated with fibrotic changes. Specifically, we found that while CNAs are common in liver tumors in both mice and humans, they are very infrequent in the nontumor cirrhotic or fibrotic liver in both species, suggesting that CNAs are not a feature of precancerous liver tissue. Cirrhosis and fibrosis, the result of chronic inflammation and subsequent myofibroblast activation, are considered to be involved in the promotion of carcinogenesis 40. Changes in the composition of the extracellular matrix and in nonparenchymal cellular activity lead to the promotion of growth and inhibition of apoptosis in the hepatic cellular environment. The profound lack of CNAs in the nontumorous fibrotic liver tissue in both human and mouse samples, a pretumorous stage of liver disease 41, and in cirrhotic liver tissue in humans, is a novel finding. If CNAs do, in fact, exist in the surrounding fibrotic or cirrhotic tissue of the liver, perhaps the accumulation of the CNAs is contingent upon clonal expansion and increased genomic instability in tumor tissue.

In relation to our previous findings of epigenetic alterations in both tumor and fibrotic surrounding tissue, as well as a lack of mutations commonly observed in HCC in both humans and mice in the same mouse tissues discussed herein 12, we posit that epigenetic alterations indicative of genomic instability precede CNAs. Further, these findings suggest that CNAs occur relatively early in the carcinogenic process compared to common activating or inactivating mutations, which is consistent with other reports of chromosomal instability as an early event in tumorigenesis 17, 38. In concordance with the findings of a recent study that evaluated the association between chromosomal instability and global DNA hypomethylation in human HCC samples 42, we observed CNAs in the same tissues in which epigenetic alterations that are indicative of genomic instability were observed. Although the mechanistic link between these two phenotypes is not clear, one potential explanation that has been proposed suggests that activation of hypomethylated repetitive DNA elements within genes may cause chromosomal rearrangements 43, a supposition that is further supported by the fact that both chromosomal instability and epigenetic alterations are generally considered early events in carcinogenesis 12, 17, 38.

An important outcome of the present study is the demonstration of the relevance of the mouse fibrosis‐associated liver cancer model to human HCC. Although the liver is the most common tissue for tumor development in experimental rodent studies of chronic exposure to xenobiotics 44, most chronic rodent cancer studies fail to induce liver fibrosis or cirrhosis, which is the most common histopathological feature observed in HCC patients and is an important mechanism of hepatocarcinogenesis 3. In the present study, many segments with altered copy number, as well as the genes within those segments, were observed in tumors from both mouse and human HCC. Mouse models that employ chemically induced carcinogenesis display a varying degree of similarity to human disease and may be well (or poorly) correlated with specific human etiologies, risk factors, or subclasses. The mouse model used herein has been reported to share many of the characteristics generally observed in cirrhosis‐associated human HCC, including, but not limited to, inflammatory response and fibrogenesis 11. Up‐regulation of mRNA markers of inflammation and fibrosis have been previously reported in the fibrotic nontumor tissues 11, 45, as well as in tumor tissue in the present study (*Tnf*,* Tgfβ1, Tgfbr1*, and *Tgfbr2*). The human samples included in the present study had various etiologies of HCC, including hepatitis B and C infection, nonalcoholic fatty liver disease, and alcohol consumption. These etiological factors, among other clinical data, are detailed in Table S3. We used all of the TCGA data available at the time that met our inclusion criteria described in Materials and Methods, which included various etiologies. It is possible that in a larger study conducted in the future, we may be able to compare our mouse model to a specific subcategory of human HCC. In the present study, the CNA profile for HCC occurring in a background of fibrosis or cirrhosis, regardless of etiology, was of primary interest.

The next important outcome of this work is in the examination of the concordance among CNAs and gene expression. Altered gene expression has been previously shown to occur in approximately half of the genes with CNAs in human cancer studies, both in clinical samples 46, 47 and in in vitro studies with immortalized human cell lines 48. These results, together with findings in our study, indicate that CNAs can and do alter gene expression, but that this particular mechanism of altered gene expression is tumor‐ and tissue‐type specific. A study of human breast tumors 46 concluded that approximately 12% of variation in mRNA levels was directly attributed to CNAs, although the authors posited that their finding “represents a significant underestimate” due to the conservative nature of the analysis, global variation in tumor cells, and the presence of nontumor cells in the samples. We found that 38% of the genes we evaluated in the mouse tumors were significantly differentially expressed in the associated direction of a copy number‐altered segment. However, we found a higher concordance between differentially expressed genes and CNAs in the human cirrhosis‐associated tumors (52% for both gain and loss). This discrepancy between species is likely due to the much more comprehensive analysis that was possible for the human samples, afforded by the RNA‐sequencing data available through TCGA. The enrichment of mitosis and cell cycle genesets is correlative with increased proliferation in the tumor cells, and the loss of immune response and cellular defense genes observed in the present study has also been previously reported in HCC, particularly in cases of viral hepatitis 49. The overrepresentation of cell cycle process genesets corroborates with the increased proliferation expected in the scenario of angiogenesis, although it cannot be concluded with certainty that these genesets are enriched due to their association with increased or decreased copy number, or if the CNAs are due to the enrichment of these mitosis‐related genesets. An increase in cell cycle processes in conjunction with chromosomal instability may represent a driver in the excessive CNAs observed in these tumors.

In a recent review of genetic alterations in HCC 50, “telomere maintenance” was identified as one of the driver pathways recurrently altered in HCC. This pathway includes *TERT* (telomerase reverse transcriptase), which is the most frequently mutated gene in human HCC, has been shown to be amplified in DNA copy number in HCC and is dysregulated in cirrhotic liver. In our study, *TERT* was increased in copy number in the human tumor samples, and the gene expression was also significantly up‐regulated. In the mouse samples, however, the *Tert* gene was not increased in DNA copy number, suggesting that this gene is more subject to CNA in humans than in mice.

It is essential to consider various mechanisms of gene regulation when evaluating the role of specific genes in cancer and when making assumptions based on copy number. For example, *Vegfa* is a gene commonly up‐regulated in cancer and was present in a frequently gained segment in both mouse and human HCC in our sample sets. However, *Vegfa* gene expression was actually significantly down‐regulated in the mouse tumor samples. This loss of expression of *Vegfa* is in contrast to previous reports of DEN‐induced mouse liver tumors 51, and could be explained by the up‐regulation of *Tgfb1* observed in our study, a gene that has been shown to inhibit *Vegfa*. *Tgfb1* is also located in a segment of copy number gain in the mouse tumors, and the mRNA level was significantly increased in the mouse tumors. Further, *Vegfa* was found to be up‐regulated in nontumor surrounding liver tissue in the mouse samples, alternatively suggesting that the expression of this gene is not directly controlled by CNA copy number in this mouse model (data not shown). Considering the number of genes that were regulated in a manner opposite of DNA copy number, many such complexities may exist in both species.

The limitations of this study should be noted. While it is widely accepted that HCC is a heterogeneous cancer that presents diverse molecular profiles across patients (or animals), as well as within an individual (or animal) in some cases 50, 52, genomic DNA from only one tumor from each mouse was used in our study. Tumor heterogeneity is thought to occur from the same selective processes that lead to common CNAs 18, 53: cells with genomic imbalances proliferate and accumulate, they acquire new mutations and CNAs 37, and clonal expansion may occur at slightly different points in the evolution of that clonal cell line. Interpatient, as well as interanimal, heterogeneity was of less concern because the goal of the present study was to find the most common CNAs among a set of tumors that were assumed to have some level of heterogeneity. Regarding the human HCC data, similar to the above‐mentioned rationale, while interpatient heterogeneity is extremely important, this study aimed to identify common CNAs among HCC patients with fibrosis and cirrhosis.

In conclusion, our results demonstrate an accumulation of CNAs in liver tumors in a mouse model of fibrosis‐associated HCC, a finding that, together with previous results demonstrating increased epigenetic alterations indicative of genomic instability, suggests that chromosomal instability is a feature of tumor cells that precedes common mutations. CNAs were more prevalent in tumors compared to surrounding fibrotic or cirrhotic liver tissue in both mice and humans, indicating that chromosomal instability is a feature specific to neoplastic rather than preneoplastic tissue in cases of HCC. Approximately half of the frequently gained or lost segments observed in the mouse fibrosis‐associated liver tumors and approximately one third of the copy number‐altered genes were also observed in human cirrhosis‐associated HCC samples. This demonstrates the similarity in genomic features between this mouse model of HCC and that of liver tumors in human patients with cirrhosis‐associated HCC.

## Conflict of Interest

The authors have no conflicts of interest to declare.

## Supporting information


**Table S1.** Selected genes and their Applied Biosystems qRTPCR primer assay numbers.

**Table S2.** Significantly enriched genesets (FDR < 0.1) in human cirrhosis‐associated liver tumors. Gene set association was analyzed using GSAASeqSP software and Molecular Signature Database curated gene ontology genesets. Genesets with bold text are present in both lists of enriched genesets: enrichment among all genes, as well as among genes that are present in a copy number segment of concordant direction. The “size” column includes how many genes are included in each geneset.

**Table S3.** Clinical data for human HCC cases. Data were downloaded from TCGA online database for the cirrhosis‐associated HCC data utilized for both copy number and gene expression analyses.
