# Supplementary material for: Characterization of copy number alterations in a mouse model of fibrosis‐associated hepatocellular carcinoma reveals concordance with human disease
Source: Cancer Med. 2016 Jan 18;5(3):574–85. doi: 10.1002/cam4.606 (PMC4799957; doi:10.1002/cam4.606)
Supplement: Supplementary file 1 — Table S1. Selected genes and their Applied Biosystems qRTPCR primer assay numbers. [file CAM4-5-574-s001.docx]

Supplementary Table 1. Selected genes and their Applied Biosystems qRTPCR primer assay numbers.

| **AppliedBiosystems Assay ID** | **Gene Symbol** | **Gene Name** |
| --- | --- | --- |
| Mm01331626_m1 | *Akt1* | thymoma viral proto-oncogene 1 |
| Mm00658541_m1 | *Erbb2* | v-erb-b2 erythroblastic leukemia viral oncogene homolog 2, neuro/glioblastoma derived oncogene homolog |
| Mm00435123_m1 | *Gadd54b* | growth arrest and DNA-damage-inducible 45 beta |
| Mm01197698_m1 | *Gusb* | glucuronidase, beta |
| Mm00492767_s1 | *Jrk* | Jerky |
| Mm00436264_m1 | *Rara* | Retinoic acid receptor, alpha |
| Mm00451387_m1 | *Spats1* | spermatogenesis associated, serine-rich 1 |
| Mm00441724_m1 | *Tgfb1* | Transforming growth factor beta 1 |
| Mm00436971_m1 | *Tgfbr1* | Transforming growth factor beta receptor 1 |
| Mm00436978_m1 | *Tgfbr2* | Transforming growth factor beta receptor 2 |
| Mm00443258_m1 | *Tnf* | Tumor necrosis factor |
| Mm01281449_m1 | *Vegfa* | vascular endothelial growth factor A |
| Mm01300555_g1 | *Wnt1* | wingless-related MMTV integration site 1 |
| Mm00442104_m1 | *Wnt10b* | wingless-related MMTV integration site 10b |
| Mm00494229_m1 | *Xrcc1* | X-ray repair complementing defective repair in Chinese hamster cells 1 |
